# Experimental Verification of the Model for Estimating the Corrosion Current of Reinforcement in an RC Element

**DOI:** 10.3390/ma18132945

**Published:** 2025-06-21

**Authors:** Faustyn Recha, Wioletta Raczkiewicz, Kamil Bacharz, Artur Wójcicki, Petra Bujňáková, Peter Koteš

**Affiliations:** 1 Faculty of Architecture, Civil Engineering and Applied Arts, Academy of Silesia, Rolna Street 43, 40-555 Katowice, Poland; 2 Department of Strength of Materials and Building Structures, Faculty of Civil Engineering and Architecture, Kielce University of Technology, Al. 1000-lecia PP 7, 25-314 Kielce, Poland; wiolar@tu.kielce.pl (W.R.); arturw@tu.kielce.pl (A.W.); 3 Department of Strength of Materials and Building Diagnostics, Faculty of Civil Engineering and Architecture, Kielce University of Technology, Al. 1000-lecia PP 7, 25-314 Kielce, Poland; kbacharz@tu.kielce.pl; 4 Department of Structures and Bridges, Faculty of Civil Engineering, University of Žilina, Univerzitná 8215/1, 010 26 Žilina, Slovakia; petra.bujnakova@uniza.sk (P.B.); peter.kotes@fstav.uniza.sk (P.K.)

**Keywords:** deflection, reinforcing steel, RC corrosion, corrosion current, durability of structures, diagnostics of RC structures

## Abstract

This article includes tests of the deflection and load-bearing capacity of reinforced concrete beams exposed to chloride ions. This work forms part of the verification of a newly developed model for estimating the intensity of the corrosion current of the reinforcement, which assumes the possibility of estimating the corrosion parameter based on a detailed analysis of the element deformation. The model assumes the use of the inverse problem, which is based on the analysis of deflection as a result of the partial impact of the corrosion process on the main reinforcement in the reinforced concrete element. This article presents, in detail, the course of the conducted tests, including the results of the deflection measurements with simultaneous measurements of the intensity of the corrosion current of the reinforcement during the test. As part of this research, a gravimetric analysis of the loss of reinforcement mass caused by the ongoing corrosion process was also performed. The main objective of this research was to experimentally verify the adopted model of the new diagnostic method, which fully confirmed the model assumptions. The obtained research results confirmed the validity of the assumptions adopted in the theoretical model, which was further confirmed by analytical calculations.

## 1. Introduction

The corrosion of the reinforcement in reinforced concrete (RC) initiated by chloride ions is an issue that has been addressed by a wide range of researchers and described in numerous publications [1,2,3,4,5,6] and scientific monographs [7,8,9]. The reinforcement corrosion process leads to the degradation of structural elements, which results, among other things, in the cracking of the cover [10,11,12,13,14], a change in the adhesion forces between steel and concrete [15,16,17,18], and a reduction in the mechanical parameters of reinforcing steel (mainly yield strength and Young’s modulus) [19,20,21,22,23,24,25,26]. The progressive degradation of the reinforcement structure and concrete during the corrosion process causes a decrease in the load-bearing capacity and safety of structural elements [27,28], as well as the size of their deflection [29,30] or the displacements of individual structure nodes [31,32]. A closely related result of damage to a reinforced concrete structure is the increase in the maintenance costs of the facility [33,34], which clearly justifies the need to undertake research related to corrosion issues. According to de Sitter’s law, the operating costs, *k*, depend on the type of damage occurring and increase abruptly via an exponential function $k=5^{x}$, where *x* is the next increase in the damage occurring [33]. This means that in a situation where corrosion processes are initiated at the moment of the depassivation of the reinforcement surface, but where surface protection is applied at this stage, the operating costs increase five times in relation to the initial operating costs, *k*. When corrosion damage to the reinforcement occurs locally, the costs increase 25 times due to local repairs to the structure or the need to apply additional protection. In extreme situations, when no protection and repairs are applied, which leads to general corrosion of the reinforcement, a complete renovation or replacement of individual structural elements may be necessary. The costs of maintaining the facility in such a situation may be as much as 125 times higher than the planned initial costs, *k*, of maintaining the facility. Ultimately, the progressive corrosion process causes damage to engineering structures such as bridges and viaducts, which are particularly exposed to the effects of chloride aggression from deicing salts during winter road maintenance.

One of the corrosion parameters that is of key importance during the analysis of corrosion damage to a reinforced concrete element is the density of the corrosion current of the reinforcement. Current intensity measurements require the use of specialist equipment and are conducted in a point-by-point manner. An alternative to this approach is a method based on estimating the corrosion current based on the analysis of the element deformation. An issue related to the corrosion problem is the measurement of the size of damage caused by the progressive corrosion of the reinforcement and the concrete structure within the corrosion center [29]. Previous analyses covering deflection issues were undertaken only with respect to increasing deflection during the development of the electrode process on the reinforcement surface [29,31]. The essence of the new method is an attempt to use the inverse problem. The assumption is based on a detailed analysis of the causes of the resulting deflection and an attempt to estimate the corrosion parameter as one of the causes of the resulting deflection. This method is based solely on deflection measurements, which makes it a completely non-invasive method. The presented solution is an auxiliary and complementary element to the previously used specialist diagnostic methods for assessing the corrosion of the reinforcement in reinforced concrete and does not eliminate them. The theoretical analysis of the proposed approach, together with numerical verification, was presented in [29], which was based on the results of measuring the operating current intensity [11]. The developed theoretical model was used to perform analytical calculations determining the course of changes in the corrosion current density of sample structures. The analyses performed are based solely on theoretically adopted assumptions, while in this article, an attempt was made to empirically verify the theoretically developed method. The verification carried out confirms the compliance of the obtained results with the point measurement results obtained with the specialist diagnostic method, which was performed on the basis of the galvanostatic electrochemical polarization pulse measurement. This is an original element of this work, not published before, and, at the same time, crucial to the diagnostic tests of structures in real-world conditions. This verification is the next component of the broader development of a new method for estimating the intensity of the corrosion current of the reinforcement in a reinforced concrete element.

## 2. Theoretical Model

The deflection of reinforced concrete elements is a coupled problem involving mechanical phenomena, cracking, concrete rheology, and progressive corrosion processes. The effects of deflection related to the passage of time include concrete creep and gradual loss of reinforcement mass [28,29], together with a gradual decrease in Young’s modulus [26,27,29]. The key issue is to divide the analysis into two phases of the structure’s performance. The first phase concerns the range of the element’s deflection before cracking, while the second phase covers the element’s performance from the moment when cracking first occurred. In the generalized state, the deflection of a reinforced concrete element is described by the following relationship [35]:

(1) $$\alpha =\zeta \cdot \alpha_{II}+(1-\zeta )\cdot \alpha_{I}$$

where α is a generalized dimensionless parameter of the considered deformation, while the coefficients $\alpha_{I}$ and $\alpha_{II}$ are the deformation values for the first and second phases of the cross-section operation, respectively. The parameter ζ takes into account the stiffening in tension analogously to the parameter known from damage mechanics:

(2) $$\zeta =\left\{\begin{array}{cc} 0 & the\;first\;phase\;of\;work\;(uncracked\;cross-section)\\ 1-\beta \cdot {\left(\frac{\sigma_{sr}}{\sigma_{s}}\right)}^{2} & the\;second\;phase\;of\;work\;(cracked\;cross-section) \end{array}\right.$$

where $\sigma_{s}$ is the stress in the tensile reinforcement at the moment of full cracking, while $\sigma_{sr}$ is the stress in the tensile reinforcement at the moment of full cracking caused by the load causing the first crack. The parameter β is a factor taking into account the time and nature of the interactions. It is β = 1.0 for a single short-term load and β = 0.5 for long-term loads, and the loads are repeated many times [35].

**Figure 1:**
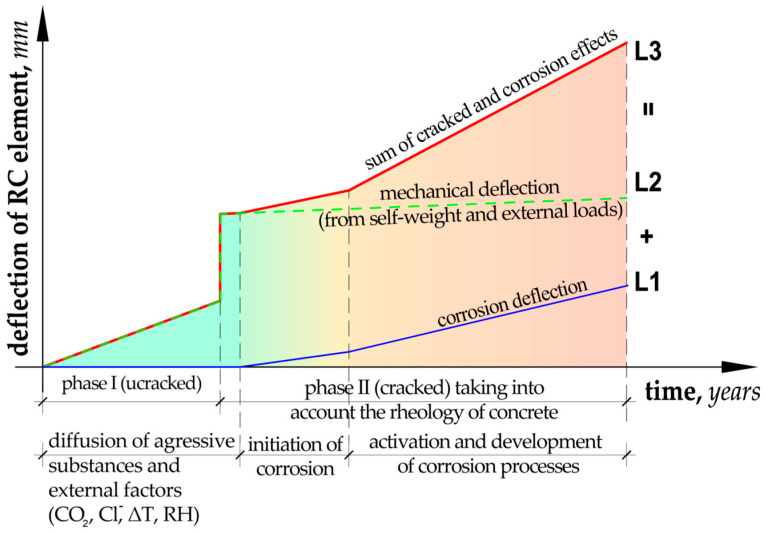
Diagram of the course of changes in the deflection of a reinforced concrete element over time.

Parallel to the mechanical effects, the impact of external environmental aggression begins, including the diffusion of factors into the concrete cover causing the initiation of the corrosion process on the reinforcement bars. The issue of moisture, heat, and aggressive substance migration has been discussed in detail in other research works, including [1,2,3,4,5,6,7,8,9], and is therefore not the subject of the analysis included in this article. In the model assumptions, it was assumed that the element cracks shortly after its loading and simultaneously before the initiation of the corrosion processes (the time necessary for the transport of aggressive substances and the penetration of the passive layer of the reinforcement). The scheme of deflection changes over time assumed in the theoretical model is presented graphically in Figure 1.

The proposed model takes into account two phases of the structure operation with simultaneous impact of the external environment on steel and concrete. The corrosion phenomena of reinforcement, including the loss of reinforcement mass and changes in Young’s modulus, are marked with a blue line (L1). The increase in deflection due to mechanical impacts (external loads and dead weight), including the creep phenomenon, is marked with a green line (L2) in the diagram. Finally, after summing up the effects of impacts, using the superposition principle, the total deflection of the element, including deflections due to mechanical impacts, was determined (red line (L3) in the diagram). According to the proposed model, the total evolution of deflection changes in the first part coincides with the course of deflection due to mechanical impacts. At the moment when corrosion initiation begins, the deflections gradually increase with the progressing corrosion process. The theoretical assumptions adopted in this way are the basis for deriving the functional relationships describing the analytical approach to estimating the corrosion current density.

## 3. Calculation Approach

According to the assumption of the model and the analysis of the deformation of reinforced concrete elements, it is possible to demonstrate the relationship between the progressive deflection of the element over time and the development of electrochemical processes on the reinforcement surface. The basis for the analysis is the history of deflection changes over time based on very precise deflection measurements or continuous monitoring of deformation. The measurement results allow for the determination of the actual stiffness of the element, $B_{corr}(t)$, and its relationship with the curvature, k, of the deflected element:

(3) $${B_{corr}(t)=I}_{corr}(t)\cdot E_{corr}(t),\;\;k=\frac{1}{r}=\frac{M_{Ek}}{B_{corr}(t)}$$

where $I_{corr}\left(t\right)$ and $E_{corr}(t)$ are the time-varying modulus of the inertia of the RC cross-section and the Young’s modulus, respectively; r is the bending radius of the element; and $M_{Ek}$ is the characteristic value of the bending moment (constant value, invariant in time). After taking into account the progressive curvature of the corroded element (3), the generalized form of the corrosion current density $i_{corr}(t)$ as a function of time increase, ∆t, derived using Faraday’s law, will take the following form [30]:

(4) $$i_{corr}(t)=∆t\left(\frac{M_{Ek}}{{∆k_{I}{(t)\cdot E}_{c.eff}(t)\cdot y}^{2}}+\frac{M_{Ek}}{∆k_{II}{(t)\cdot E}_{c.eff}(t){\cdot y}^{2}}\right)\frac{\rho_{Fe}\cdot l_{eff}}{k_{eff}\cdot A_{b}}$$

where $∆k_{I}(t)$ and $∆k_{II}(t)$ are the values of curvatures for the first and second phase of the structure operation, respectively, after the time, ∆t; y is the distance from the center of gravity to the extreme tensile fibers of the cross-section; $\rho_{Fe}$ is the density of the reinforcing steel; $l_{eff}$ is the effective length of the element [35]; $k_{eff}$ is the effective electrochemical equivalent of the reinforcing steel designated according to [11]; and $A_{b}$ is the reinforcement surface area where electrochemical processes develop (it is not the reinforcement cross-section area). The Young’s modulus of concrete $E_{c.eff}(t)$ in Equation (4) takes into account the effects of concrete rheology based on the creep coefficient, $\varphi \left(\infty ,t\right)$, and the mean secant Young’s modulus, $E_{cm}$ [35,36]:

(5) $$E_{c.eff}=\frac{E_{cm}}{1+\varphi \left(\infty ,t\right)}$$

The assumptions of the theoretical model (Figure 1) show that the initiation of the corrosion process occurs after the structure is cracked (in the second phase of operation). Therefore, Equation (2) will be reduced to the following form:

(6) $$i_{corr}(t)=∆t\left(\frac{M_{Ek}}{∆k_{II}{(t)\cdot E}_{c.eff}(t)\cdot y^{2}}\right)\cdot \frac{\rho_{Fe}\cdot l_{eff}}{k_{eff}\cdot A_{b}}$$

The increase in curvature after cracking $∆k_{II}(t)$ refers to the moment of inertia of the entire cross-section in the cracked state $∆I_{cr}$. The corrosion effects are taken into account by relating the inertia modulus $∆I_{cr}$ to the reinforcement cross-section area $∆A_{s}$, changing due to the loss of reinforcement mass. This is possible by applying Faraday’s second law:

(7) $$∆m=\rho_{Fe}\cdot {∆V}_{s}=\int_{0}^{t}{(k}_{eff}\cdot i_{corr}{\cdot A}_{b})dt,\;\;{∆V}_{s}={∆A}_{s}\cdot l_{eff}$$

The effective surface area of the corroded reinforced concrete element $A_{corr}$ can therefore be defined as the sum of the surface areas of the concrete $A_{c}$ and the changing reinforcement ${∆A}_{s}$.

(8) $$∆A_{corr}=A_{c}+\alpha_{e}\cdot {∆A}_{s},\;\;\alpha_{e}=\frac{E_{s}(t)}{E_{c,eff}}$$

where $E_{s}\left(t\right)$ is the variable Young’s modulus of the reinforcing steel due to the progressive corrosion of the reinforcing steel. Then the definition of the modulus of inertia was used, as the product of the cross-sectional area, $∆A_{corr}$, and the square of the distance from the center of gravity to the extreme tensile fibers$,\;y^{2}$. After substituting it into Equation (3) and rearranging it, it is possible to define the reduced cross-sectional area $∆A_{corr}$ based on the curvature of the element, which decreases with the progressing corrosion process:

(9) $$∆A_{corr}=\frac{∆I_{corr}}{y^{2}}=\frac{M_{Ek}}{∆k_{II}(t){\cdot E}_{c,eff}{\cdot y}^{2}}$$

After substituting Equation (9) into Equation (8) and transforming, the magnitude of the change in the reinforcement cross-sectional area$,\;{∆A}_{s}$, as a function of the element curvature is obtained:

(10) $$∆A_{s}=\left(\frac{M_{Ek}}{∆k_{II}{(t)\cdot E}_{c,eff}{\cdot y}^{2}}-A_{c}\right)\frac{1}{\alpha_{e}}$$

After transforming expression (6), and taking into account the changes in the reinforcement cross-sectional area$,\;∆A_{s}$, according to Equation (10), the value of the corrosion current density as a function of time $i_{corr}(t)$ takes its final form, which is mainly based on the analysis of the changes in the curvature after scratching the $∆k_{II}(t)$ of the element deflection axis and taking into account the creep and changes in the reinforcement Young’s modulus as a function of time:

(11) $$i_{corr}(t)=\frac{\rho_{Fe}\left[\left(\frac{M_{Ek}}{∆k_{II}{(t)\cdot E}_{c,eff}{\cdot y}^{2}}-A_{c}\right)\frac{1}{\alpha_{e}}\right]l_{eff}}{k_{eff}{\cdot A}_{b}\cdot ∆t}$$

## 4. Laboratory Tests

**Figure 2:**
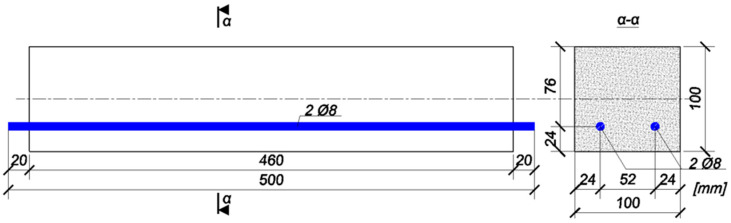
Geometry of the test beam subjected to laboratory tests.

The tests were carried out on four beams with dimensions of 100 × 100 × 460 mm, reinforced at the bottom, with two bars of 8 mm in diameter and 500 mm in length (the free ends of the reinforcement extended beyond the concrete outline by 20 mm). The reinforcement cover was the same in all cases and amounted to 20 mm (Figure 2).

The concrete mix for the beams was made of sand (fraction 0–2 mm), washed aggregate with granulation 2–8 mm, and Portland cement CEM I, 42.5 N. The concrete mix was made in the ratio 0.54/1.00/1.23/4.92 (water/cement/sand/aggregate). At the stage of preparing the concrete mix, chlorides ions ${Cl}^{-}$ were added to the mixing water at a concentration of 6%. Most standards for testing the durability of structures with the inclusion of de-icing agents assume a level of 3%. For the purposes of this work, the possibility of a locally occurring concentration twice as high was assumed. After demolding the beams and test samples for strength tests, these elements were left for a maturing period of 28 days also in a 6% NaCl solution. Then, cubic concrete samples with dimensions of 150 × 150 × 150 mm were subjected to uniaxial compression tests in a testing machine. On this basis, it was determined that the concrete of the beams was class C40/50 [37]. The results of the compression tests of the concrete samples are included in Table 1.

The reinforcement used was class C reinforcing steel, grade B500SP, with a characteristic yield strength of 500 MPa and the chemical composition given in Table 2. Ribbed, hot-rolled steel of increased quality of workmanship, marked with the EPSTAL quality mark, was used. The product has strength and deformation parameters of high stability. It has very good adhesion to concrete and is manufactured in a certified plant where a quality control system has been implemented. No other reinforcement was intentionally placed in the beams to avoid constraining the structure. This research focused exclusively on the corrosion of longitudinal bars in the tension zone, which are responsible for the deflection effect.

**Table 2:** Chemical composition of B500SP reinforcing steel as declared by the manufacturer.

Assay	Maximum Content of Elements, %							$C_{eq.max}$%
	C	Mn	Si	P	S	Cu	N	
steel smelting	0.220	1.600	0.550	0.050	0.050	0.800	0.012	0.500
finished product	0.240	1.650	0.600	0.055	0.055	0.850	0.013	0.520

Notes: C—carbon; Mn—manganese; Si—silicon; P—phosphorus; S—sulfur; Cu—copper; N—nitrogen; $C_{eq.max}$—carbon equivalent. According to (12): (12) $$C_{eq.max}=C+\frac{Mn}{6}+\frac{\left(Cr+V+Mo\right)}{5}+\frac{\left(Cu+Ni\right)}{15}$$

**Figure 3:**
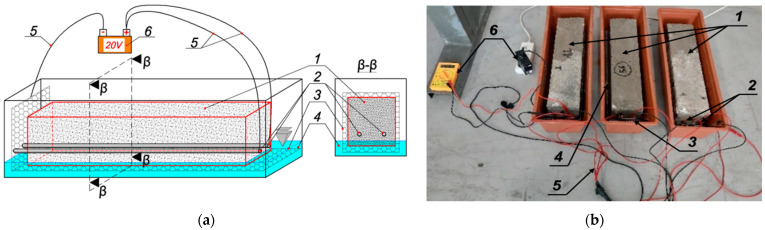
Simulation of the corrosion process using an electrolyzed system (description in the text): (**a**) diagram; (**b**) laboratory stand.

The simulations of the corrosion process were carried out in two stages. The first stage included adding 6% ${Cl}^{-}$ chlorides to the mixing water at the time of preparing the concrete mix (uniform distribution of chloride ions in the mix and then in the concrete structure). Then, after 24 h, the beams were dismantled and placed in brine of the same concentration to prevent chloride diffusion outside the element [38]. The maturation period of the beams in this environment was 12 months. In the second stage, beam 1 was subjected to corrosion simulation in an accelerated corrosion test using an electrolyzer system, in which the beam reinforcement was cathode 2, while the external sheet was anode 3 (Figure 3). A current of 20 V was applied to the entire system using copper wires in silicone cover 5 and maintained for 62 h by an external power supply 6. The water level 4 in the vessel reached only the height of the reinforcement cover (~20 mm) to minimize the uncontrolled current flow outside the concrete area (through the reinforcement bars protruding from the beams). In the second stage, the corrosion process was accelerated by means of an electrolyzer system, where the anode electrode was placed along the reinforcement so that the current flow was uniform. The effect of such a phenomenon could be the dissolution of steel in the zone where it does not affect the subject of the tests. The development of corrosive processes within the concrete structure is important [11].

**Figure 4:**
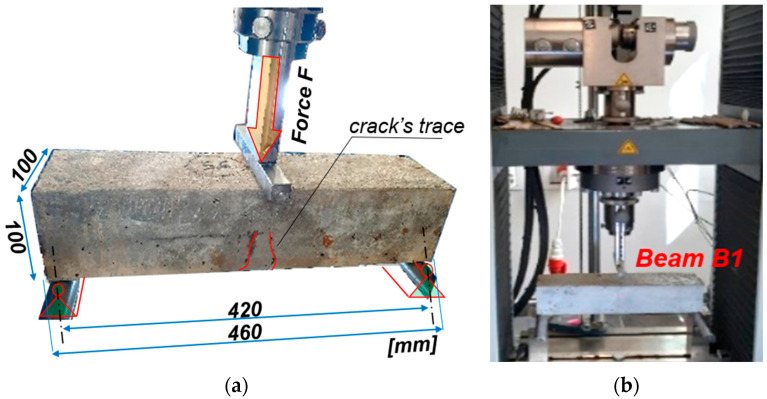
Test stand with a sample ready for testing: (**a**) static diagram; (**b**) view of sample B1 in the testing machine.

The electrode process was interrupted for the purpose of performing load tests, optical measurements, and tests of the actual corrosion current intensity level, which were performed in three subsequent attempts by gradually increasing the pressure force on the beam (Figure 4).

**Figure 5:**
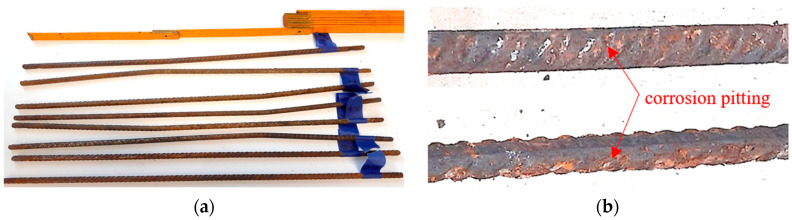
Bars subjected to analysis after splitting the samples and performing (**a**) gravimetric analysis and (**b**) macroscopic analysis. There are visible corrosion pits on the reinforcement surface on the example of bar $P_{1}$.

In the tests, the electrochemical polarization galvanostatic pulse method and the GP-5000 Galva Pulse ^TM^ measuring set [39] were used to measure the corrosion current intensity. The first current intensity measurement (reference) was taken immediately before the accelerated corrosion test. The next measurement was taken after approximately 30 h, and the next one after 32 h. After the tests, the samples were split, and the reinforcing bars were removed and cleaned of loose corrosion products. Macroscopic analysis showed the formation of corrosion pits on the reinforcement surface, which is consistent with the damage mechanism resulting from chloride corrosion [40] (Figure 5). Detailed microscopic analysis of the reinforcement damage surface, including atomic analysis of the oxide layer, was performed in another article, where a detailed analysis of the changes in the mechanical properties and the damage of the same reinforcing steel was undertaken.

The bars were then subjected to gravimetric analysis to determine the actual reinforcement mass loss. The list of test samples, along with the gravimetric analysis results for all beams, is presented in Table 3.

The loss of mass of the bars was determined based on the difference between the initial mass $m_{0}$ and the mass $m_{t}$ weighed successively after the bars had been derusted in phosphoric acid and mechanically cleaned. The difference in measurements, ∆m, was the actual loss of mass of the reinforcement, which was also converted into a percentage loss $∆m_{p}$ and a degree of corrosion $\alpha_{corr}$:

(13) $$∆m_{i}=m_{0,i}-m_{t,i}$$

(14) $$∆m_{p,i}=\frac{m_{0,i}-m_{t,i}}{m_{0,i}}\cdot 100\%$$

(15) $$\alpha_{corr.i}=\frac{{∆m}_{avg.i}}{m_{0.avg.i}}\cdot 100\%$$

where $∆m_{i}$ is the actual mass loss of the reinforcement, $∆m_{p,i}$ is the percentage mass loss of the reinforcement, $m_{0,i}$ is the initial mass (before the test), $m_{t,i}$ is the final mass (after the test), ${∆m}_{avg,i}$ is the ratio of the average mass loss of the reinforcement to the average initial mass $m_{0.avg,i}$, and $\alpha_{corr.i}$ determines the corrosion degree. In the next stage, using Faraday’s second law, the actual values of the electrochemical equivalent of reinforcing steel $k_{eff.i}$ were determined separately for each bar, which were then averaged for each beam and the entire test task (see Table 3).

(16) $$k_{eff.i}=\frac{∆m_{i}}{I_{corr,i}\cdot ∆t}$$

## 5. Analysis of the Obtained Results

**Figure 6:**
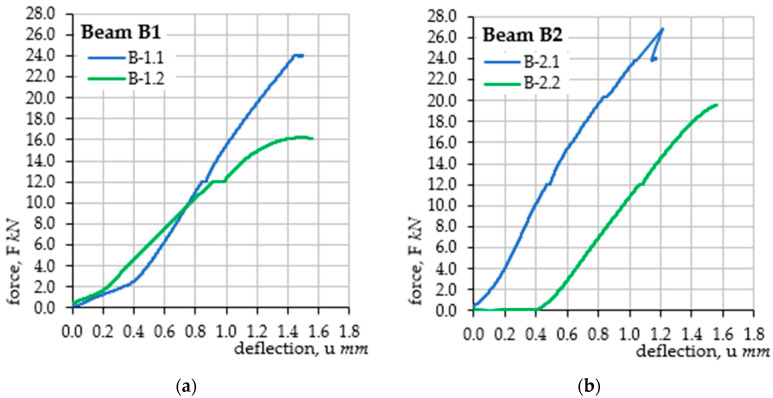

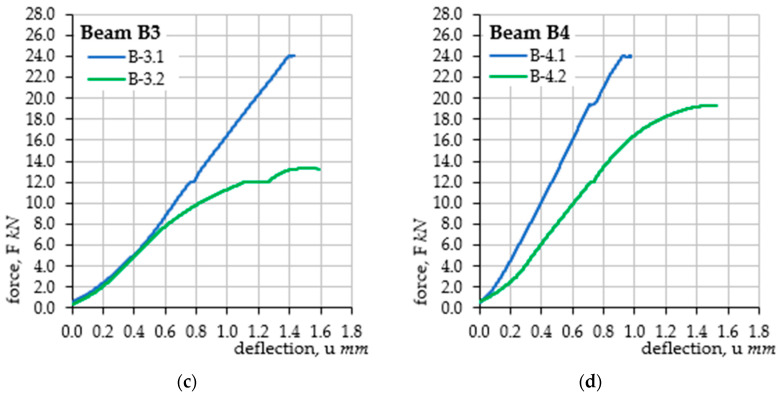
Results of beam deflection tests during the ongoing corrosion process: (**a**) sample B1; (**b**) sample B2; (**c**) sample B3; (**d**) sample B4.

As part of the conducted tests, graphs of the force F(u) relationship, i.e., the force-deflection of the beam, were obtained. Analysis of the mechanical changes of the beam was performed in real time, continuously monitoring the increase in force, *F*, and the corresponding deflection, *u*. The tests were conducted on an electromechanical testing machine Zwick Roell Z250, which allows for testing in a load range from 5 kN to 250 kN, in the form of tension, compression, and bending tests. In the discussed case, in accordance with Figure 4, a three-point bending test was performed with a two-stage load at a loading speed of 0.4 kN/min. The testing machine used allows for continuous monitoring, the recording of the set load value, and the recording of the displacement of the crossbeam, which, due to the static scheme, corresponded to the deflection of the tested elements. The recording frequency was assumed to be at 5 Hz. It was assumed that the maximum vertical force applied to each beam would be equal to 24 kN. Deflections were tested in two successive stages: the first stage covered the beam immediately before the accelerated corrosion test; while the second stage was performed after 62 h of charging in the electrolyze system. During both stages, three measurements of the corrosion current intensity were performed: the first for the unloaded beam, the second when the beam was loaded with a force of 12 kN (half of the maximum force), and the third when the beam was loaded with a maximum force of 24 kN. The visible jumps in the history graphs of changes within the load value of 12 kN result from the time necessary to measure the corrosion current intensity in the middle of the load cycle. The summary of the performed deflection measurements is presented in Figure 6. As part of the verification of the method for estimating the corrosion current intensity, the results of the corrosion current intensity measurements during the tests were compared with the results obtained on the basis of analytical calculations using the proposed method for estimating the corrosion current density. The computational analysis was based on the results of laboratory tests on the concrete class, which allowed for the determination of the cracking moment,$\;M_{cr}$ = 0.583 kNm, which is the limit for the first phase of the structure’s operation (before cracking). The creep coefficient was assumed to be the same for all the analyzed samples and equal to φ = 2.5. Finally, the average value obtained for the entire research task $k_{eff.avg}$ = 0.0065178 g/(μA*year) was selected for the analytical calculations of the test elements (see Table 3). For the purposes of the calculations, a uniform decrease in the Young’s modulus of the reinforcing steel was also assumed. The Young’s modulus was assumed to decrease by 10% of the initial value in a linear function evenly during the test, namely, before the test the Young’s modulus,$\;E_{s(t=h)}$ = 200 GPa was assumed, while after the accelerated corrosion test, $E_{s\left(t=62h\right)}$ = 180 GPa. This value was assumed based on separate experimental studies [40]. As a result, a linearly varying coefficient, $\alpha_{e}$, was obtained, illustrating the ratio of the Young’s moduli of concrete and steel, which, in this study, was in the range of 〈$\alpha_{e\left(t=0h\right)}$ = 19.44; $\alpha_{e\left(t=62h\right)}$ = 17.50〉.

**Figure 7:**
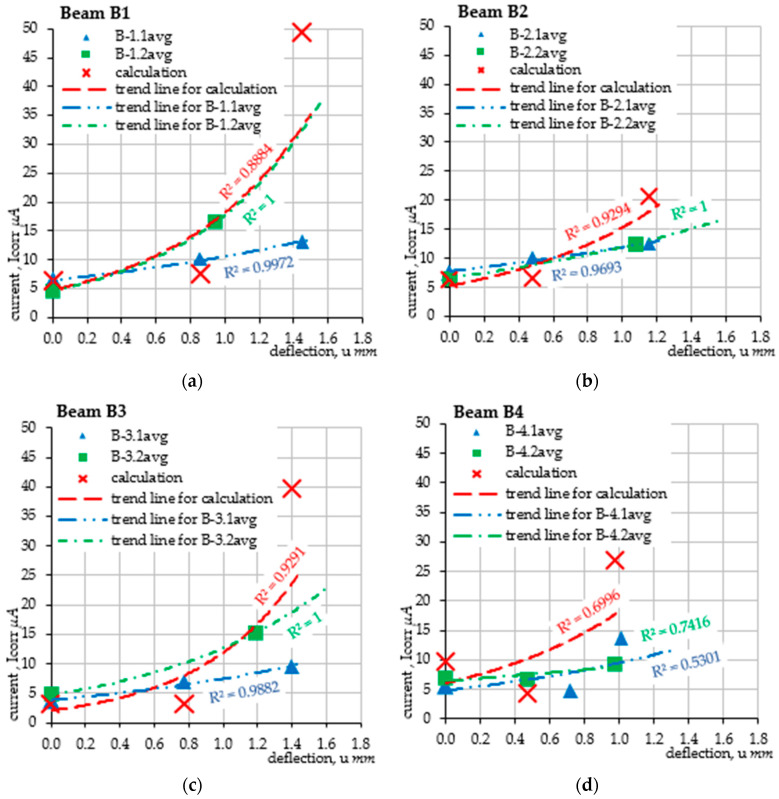
Results of the undertaken verification of the corrosion current intensity level: (**a**) sample B1; (**b**) sample B2; (**c**) sample B3; (**d**) sample B4.

The obtained results are presented in the form of scatter plots of the reinforcement corrosion current intensity measurements for the values averaged from two measurements of each beam performed for each bar separately. All results are marked with the symbols *B-i.j.avg*, where *i* denotes the beam number and *j* is the measurement number. For the same measurement points, the estimated values of the corrosion current intensity were determined analytically such that their comparison was as optimal as possible in terms of the accuracy of the obtained results. Comparison of individual results, although precise, does not provide a full picture of the parameter changes as a function of progressive deflection. For this reason, trend lines in the exponential form were determined for both the laboratory results and the calculation results. The evolution of the course of changes in the corrosion current density as a function of progressive deflection is illustrated in the graphs (Figure 7). Particularly accurate agreement of the results was obtained in the case of beam B1, for which the trend line of the calculation results practically coincides with the trend line obtained from the results of the second measurement cycle. Slightly lower accuracy was obtained for the remaining beams B2, B3, and B4, where the trend lines of the calculation results have smaller values for small deflections, while higher values for deflections that occurred during the beam destruction. The shift of the trend line in each case is caused by the simplification of the model in the form of omitting the range of the first phase of the structure’s operation due to the relatively small value of the cracking moment ($M_{cr}$ = 0.583 kNm), at which the tensile stresses in the concrete are exceeded; thus, its cracking occurs within the tensile reinforcement. The cracking moment $M_{cr}$ was obtained at a load of 21% of the breaking load (F = 24 kN). This assumption is also correct due to the cyclically performed measurements, which cause the first loading approach to cause the formation of cracks. In subsequent loads, the beam therefore works only in the second phase of the structure’s operation (after cracking). The bending moment during the measurement at the force level F = 12 kN (half of the total load) was 1.389 kNm, while in the case of the ultimate force of 24 kN, this moment was approximately 2.778 kNm.

Finally, the average values of all results obtained for individual measurements were determined, both those obtained during laboratory tests and those resulting from calculations:

(17) $$\mathrm{First}\;\mathrm{measurement}\;\left(B1.1\_B4.1\right)avg=\frac{\sum_{n=4}^{i=4}B_{i.1}}{n}\;\mathrm{Sec}\mathrm{ond}\;\mathrm{measurement}\;\left(B1.2\_B4.2\right)avg=\frac{\sum_{n=4}^{i=4}B_{i.1}}{n}\;\mathrm{Analytical}\;\mathrm{calculations}\;calculation.avg=\frac{\sum_{n=4}^{i=4}{calc}_{i}}{n}$$

**Figure 8:**
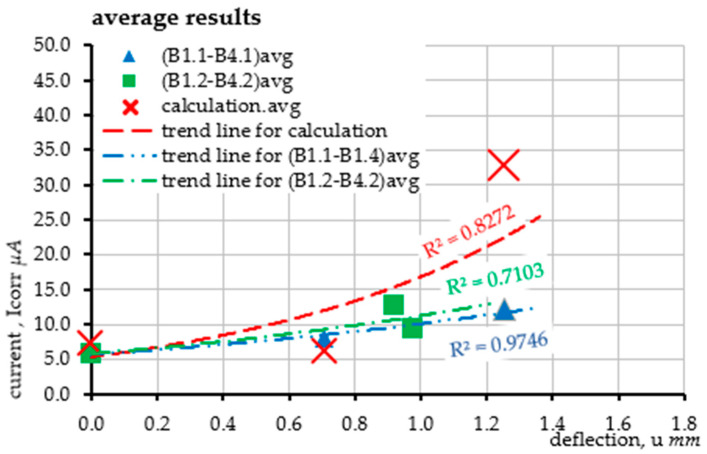
Averaged results of the undertaken verification of the corrosion current intensity level.

The obtained results of averaged values are presented in the form of a graph of the evolution of current intensity changes in correlation with the resulting deflection (Figure 8). Similarly to the case of precise results for each sample, the shape and course of the graph match the results obtained from laboratory tests, especially in the initial range of the study.

## 6. Discussion

In the work, four test samples were made and subjected to natural chloride corrosion, which was then accelerated by the electrochemical method. The tests consisted of a cyclic analysis of the deflection and load-bearing capacity of each element with simultaneous measurement of the level of the corrosion current intensity of the reinforcement. The obtained deflection results were the basis for estimating the density of the corrosion current and verifying them with the values measured during the tests. This is the first verification approach of the proposed method, which will be expanded with subsequent elements of the model, taking into account accompanying phenomena. At this stage, all beams obtained comparable results, which is the basis for further research and development of the method. The conducted experiments and detailed analysis of the results provided the basis for the following conclusions:

- The progressing corrosion process causes an increase in the deflections of the elements loaded in the same way, which is consistent with the results of the tests [23,27,29,30,31]. This is confirmed by the graphs of the evolution of changes in the displacement of the point in the middle of the span of the analyzed beams as a function of the concentrated force. The obtained deflection in the second loading cycle (after the accelerated corrosion test) is greater than the deflection in the first cycle (before the accelerated corrosion test) with the same external force (see Figure 6).
- The proposed method for estimating the level of the corrosion current intensity of the reinforcement was verified based on the analysis of beam deflections and simultaneous measurement of the actual value of the corrosion current intensity. The deflection, which was the basis for determining the curvature of the element, and then the changes in stiffness in the corroded element, provided the basis for estimating the corrosion current intensity using Faraday’s law. The obtained results were comparably similar to the results of laboratory measurements. A more accurate illustration of the obtained results was obtained based on generating exponential functions of the trend line for each series of tests. Thanks to this, the course of the evolution of changes in the corrosion current intensity as a function of the element deflection was generated using the proposed method. The graphs of the obtained functions mostly reflect the actual state of changes in the corrosion current intensity. The result that deserves special attention are the graphs obtained for beam B1 in the second load cycle, where the course of changes in the current intensity obtained in the tests has a very high compliance with the graph obtained for the proposed calculation method. In the remaining cases, the graphs are also similar but have slightly larger scatters.
- In each analyzed case, in the first range, the level of corrosion current intensity is lower than the actual values; then, it increases, finally reaching values higher than the actual state. These differences are small and result from simplifications of the theoretical model, which in the case of the estimation method is considered a fully satisfactory result. Differences in the individual stages of the impact result directly from the simplification used at the stage of performing analytical calculations, consisting of omitting the range of the first phase of the work of the bent element (until the beam cracks) due to cyclic loads of the same elements, which, after the first cycle, were plastically deformed-cracked.
- Finally, it was considered that the estimation results performed within the proposed new research method reflect the actual state of the corrosion current intensity level in the element well. The proposed method is an element supporting existing, advanced diagnostic methods, while its advantage over other methods is the absolute non-invasiveness in the structure. This method is fully based on external measurements of deflections or deformations of the structure, which must be performed with very high accuracy (including optical image analysis methods). The $R^{2}$ coefficient of determination obtained for the calculated values of the corrosion current intensity for most cases at the level of about 0.9 is considered a satisfactory result. Fitting the function with an error oscillating around 10% can be considered acceptable for the method representing the estimation approach. Only the trend line fitting in the case of beam B4 is burdened with larger errors due to the uncertainties of the model parameters that are burdened with corrosion processes. Progressive deflection is the sum of mechanical deflections (from external loads and self-weight) and rheological and corrosive phenomena. In addition, the effects of concrete cover cracking due to corrosion and changes in adhesion at the steel–concrete interface are an additional element of the model, which requires in-depth verification at later stages of its development. Therefore, an additional issue is the application of probabilistic issues, including the Monte Carlo method or interval numbers, to the assessment of phenomena burdened with large uncertainties, such as the corrosion process. Due to the complexity of corrosion issues and the mechanics of cracking the structure, which reduce the stiffness of the element, the method can only be used on the basis of precise deflection measurements. Optical measurement and image analysis methods may prove helpful in this situation (e.g., including optical methods, self-learning technology or AI). In addition, this method can be expanded to include analysis of the obtained images and the automatic determination of the corrosion rate of the reinforcement using mobile applications. This is a topic that is planned to be developed in further research.

## 7. Conclusions

As part of the verification process, we present three main conclusions:

- The corrosion process that occurs causes an increase in the deflection of the element, which is caused by the change in the stiffness of the reinforced concrete element due to the coupled phenomenon of the loss of reinforcement mass and changes in Young’s modulus. This phenomenon confirms the correctness of the assumptions made in the theoretical model, and the obtained results are consistent with similar studies in this area [29,30,31].
- The proposed theoretical method confirms the possibility of estimating the level of reinforcement corrosion current intensity based on the deflection of reinforced concrete elements in a completely non-invasive way, solely on the basis of the analysis of the deformation of the structure, which is the original element of this method.
- The presented method, together with the function describing the evolution of changes in the corrosion current in relation to the measured deflection, can be used in the diagnostics of structures for the purposes of the preliminary estimation of the corrosion level. As part of the development of the theoretical model of this method, we plan to apply solutions that take into account the uncertainty of the model parameters (e.g., the Monte Carlo method) and the influence of concrete cover cracking on deflection phenomena (including changes in adhesion forces between steel and concrete). The last element planned in the course of this research is the verification and application of this method for reinforced concrete elements operating in real-world conditions.

## Figures and Tables

**Table 1:** Mechanical properties of concrete used for beam tests.

Scenario	Sample	Dimension mm	$\mathrm{Force},\;F_{i}$ kN	$\mathrm{Stress},\;\sigma_{i}$ MPa	$\mathrm{Average}\;\mathrm{Stress},\;{\bar{\sigma }}_{l}$ MPa
Beam’s concrete	C1	150 × 150	1141.328	50.726	48.788
	C2		1122.253	49.878	
	C3		1089.341	48.415	
	C4		1038.008	46.134	

Notes: $F_{i}$ is the ultimate force; $\sigma_{i}$ is the ultimate compressive stress; $\bar{\sigma_{i}}$ is the average ultimate compressive stress.

**Table 3:** Summary of tested samples, gravimetric results, and electrochemical reinforcement equivalent results.

Scenario—Beam’s Number	Rebar’s Number	$m_{0,i}$ g	$m_{t,i}$ g	$∆m_{i}$ g	${∆m}_{avg,i}$ g	$∆m_{p,i}$ %	$\alpha_{corr,i}$ %	∆t year	$I_{corr,i}$ μA	$k_{eff.i}$ g/(μA·year)	$k_{eff.avg.i}$ g/(μA·rok)	$k_{eff.avg}$ g/(μA·year)
Beam B1	$P_{1}$	194.91	186.75	8.16	8.490	4.19	4.33	0.0082192	18.75	0.005294933	0.00641009	0.0065178
	$P_{2}$	196.88	188.06	8.82		4.48			14.26	0.007525245		
Beam B2	$P_{3}$	194.85	188.36	6.49	5.980	3.33	3.08		13.12	0.00601842	0.00582204	
	$P_{4}$	193.66	188.19	5.47		2.82			11.83	0.005625669		
Beam B3	$P_{5}$	196.26	190.49	5.77	5265	2.94	2.70		16.36	0.004291055	0.00419338	
	$P_{6}$	193.73	188.97	4,76		2.46			14.14	0.00409571		
Beam B4	$P_{7}$	194.00	187.70	6.30	5.305	3.25	2.73		6.77	0.011322009	0.00964568	
	$P_{8}$	195.16	190.85	4.31		2.21			6.58	0.007969352		

Notes: $m_{0.i}$ is the initial mass of rebar; $m_{t.i}$ is final mass of rebar; $∆m_{i}$ is mass loss; $s_{i}$ is population standard deviation$;{∆m}_{avg.i}$ is average mass loss; $∆m_{p.i}$ is percentage mass loss; $\alpha_{corr.i}$ is the corrosion degree; ∆t is the time increase; $I_{corr,i}$ is the measured corrosion current intensity; $k_{eff}$ is the electrochemical equivalent of reinforcing steel (characteristic values for individual results and average values for subsequent beams and the entire research task, respectively).

## Data Availability

The original contributions presented in this study are included in the article. Further inquiries can be directed to the corresponding author.

## Nomenclature

$m_{0.i}$	initial mass of rebar, g
$m_{t}$	final mass of rebar, g
∆m	mass loss, g
${∆m}_{avg}$	average mass loss, g
$∆m_{p}$	percentage mass loss, %
$\alpha_{corr}$	corrosion degree, %
F	ultimate force, kN
$\sigma_{s}$	stress in reinforcing steel, MPa
$\sigma_{sr}$	stress in reinforcing steel causing first crack, MPa
α	deformation parameter, -
β	factor taking into account time and nature of external impact, -
$B_{corr}$	flexural stiffness of corroded reinforced concrete cross-section, kN$\cdot m^{2}$
$I_{corr}$	modulus of inertia of corroded reinforced concrete cross-section, $m^{4}$
$E_{corr}$	Young’s modulus of corroded reinforced concrete cross-section, GPa
$M_{Ek}$	bending moment, characteristic value, kNm
k	curvature of deflection, 1/m
$M_{cr}$	critical bending moment, kNm
r	radius of deflection curvature, m
t,∆t	time, time increase, year
$\rho_{Fe}$	density of reinforcing steel, g/${cm}^{3}$
$l_{eff}$	effective length of element, m
$A_{b}$	area of side surface of bar identical to the area of electrochemically active areas, ${cm}^{2}$
$k_{eff}$	effective electrochemical equivalent of reinforcing steel, determined experimentally, g/(μA·year)
$E_{c.eff}$	Young’s modulus taking into account concrete rheology, GPa
$E_{cm}$	mean secant Young’s modulus of concrete, GPa
$\varphi \left(\infty ,t\right)$	concrete creep coefficient, -
$i_{corr}$	density of corrosion current intensity, μA/${cm}^{2}$
${∆A}_{s}$	change in cross-sectional area of reinforcement, ${cm}^{2}$
${∆V}_{s}$	change in volume of reinforcement, ${cm}^{3}$
$A_{c}$	cross-sectional area concrete, ${cm}^{2}$
$E_{s}$	Young’s modulus of reinforcing steel, GPa
$\alpha_{e}$	coefficient taking into account the ratio of Young’s moduli of reinforcing steel and concrete, -
y	distance from the center of gravity to the extreme tensile fibers of a reinforced concrete cross-section, cm
